# An accessible HPLC-DAD method for the direct detection of acrolein-trapping compounds in complex plant matrices^☆^

**DOI:** 10.1016/j.mex.2025.103747

**Published:** 2025-12-03

**Authors:** Andy Zedet, Alison Aebischer, Marc Pudlo, Luca Marchisio, Samba Fama Ndoye, Corine Girard, François Senejoux

**Affiliations:** a Université Marie et Louis Pasteur, EFS, INSERM, RIGHT (UMR 1098) F-25000 Besançon, France; b Laboratory of Organic and Therapeutic Chemistry, Faculty of Medicine, Pharmacy and Odontology, Cheikh Anta Diop University, PB 5005 Dakar-Fann, Senegal

**Keywords:** Reactive carbonyl species, Acrolein scavengers, High-performance liquid chromatography, Natural products, Plant extracts, Bioactive compounds identification

## Abstract

Acrolein is a highly reactive α,β-unsaturated aldehyde implicated in numerous diseases and pathological conditions. Developing strategies to alleviate its harmful effects is thus of key importance, with scavengers that trap acrolein emerging as a promising approach. Recent efforts have focused on identifying effective phytoconstituents, but detecting active components in complex plant matrices remains a challenging and time-consuming task. This study introduces a new application of HPLC-DAD for the instantaneous detection of acrolein scavengers in such complex extracts. To mimic this chemical diversity and test the method’s efficiency, a multicomponent mixture of ten phytochemical standards was employed. The procedure involved pre-column incubation of the mixture with varying concentrations of acrolein, allowing for the selective identification of active components through signal reduction. The results were further validated through conventional evaluation of individual constituents, confirming the method's reliability.

• Development of a novel application of HPLC-DAD for the instantaneous detection of acrolein-trapping constituents in complex plant matrices
• Application of the method to a ten-phytoconstituent mixture designed to simulate the chemical complexity of a plant extract
• Validation of method efficiency through comparison with conventional scavenging evaluations of individual compounds

## Graphical abstract

**Figure:**
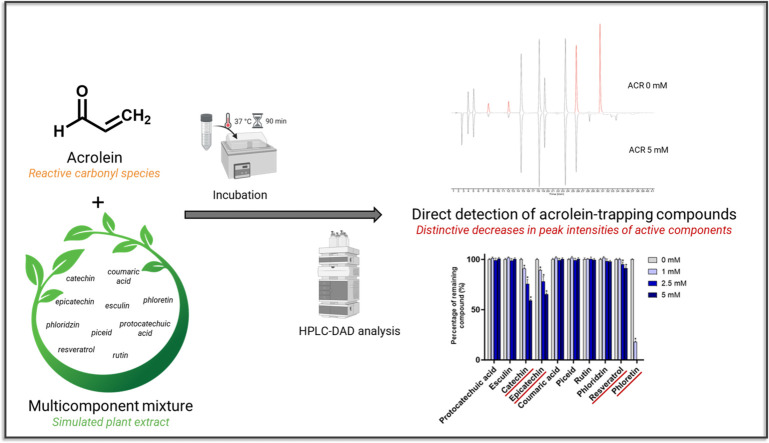

**Specifications table**

**Table:** 

**Subject area**	Chemistry
**More specific subject area**	Bioanalytical methods; Natural products chemistry; Drug discovery
**Name of your method**	A pre-column incubation approach coupled with HPLC-DAD analysis for fast screening of acrolein scavengers in complex plant extracts
**Name and reference of original method**	Pre-column incubation followed by fast liquid chromatography analysis for rapid screening of natural methylglyoxal scavengers directly from herbal medicines: Case study of Polygonum cuspidatum Dan Tang, Jia-Xiao Zhu, An-Guo Wu, You-Hua Xu, Ting-Ting Duan, Zhao-Guang Zheng, Ru-Shang Wang, Dan Li, Quan Zhu. Journal of Chromatography A, 2013, 1286, Pages 102–110, https://doi.org/10.1016/j.chroma.2013.02.058
**Resource availability**	None

## Background

Acrolein (2-propenal) is a highly toxic and reactive α,β-unsaturated aldehyde which can arise from both exogenous and endogenous sources. Diet is one of the main sources of exogenous acrolein, particularly through the consumption of fat-rich foods subjected to high-temperature processing. Acrolein is also found in environmental pollutants such as tobacco smoke and vehicle exhaust. In addition to these exogenous sources, it can be produced endogenously through various biochemical pathways, including lipid peroxidation as well as amino acid and polyamine metabolisms [1,2]. The toxicity of acrolein is primarily attributed to its strong electrophilic nature, which enables it to react with nucleophilic targets within the cell. This includes depletion of cellular glutathione (GSH) and covalent modification of macromolecules such as proteins and nucleic acids, both of which contribute to its pathogenic effects [2]. The growing body of evidence connecting acrolein to various serious diseases underscores the importance of designing strategies to mitigate its deleterious effects, with acrolein-trapping scavengers representing a particularly promising avenue [3].

Of interest, the plant kingdom has been reported as a significant source of compounds with acrolein-scavenging potential. Notably, chalcones like phloretin [4] and stilbenes such as resveratrol and piceatannol [5,6] have demonstrated effective scavenging activity. However, the number of identified bioactive compounds remains limited, highlighting the urgent need to explore and identify new, and potentially more potent, natural acrolein scavengers. It has, however, to be noted that detecting active components in complex plant matrices remains a challenging task [7]. Bio-guided fractionation might constitute an effective method, but numerous substantial restrictions arise from this time-consuming, labor-intensive and relatively expensive approach. The implementation of original strategies capable of accelerating the identification of new acrolein trapping compounds is thus of major importance. Interestingly, chromatographic protocols have recently been developed to identify carbonyl-trapping constituents in plant extracts by pre-incubating them with a carbonyl derivative before analysis [8,9]. Covalent adduct formation reduces the peak areas of bioactive compounds, allowing their distinction from inactive ones. Notably, this approach has already been successfully applied to the identification of scavengers of methylglyoxal [8,9], a reactive carbonyl species notably produced from the non-enzymatic degradation of sugars. However, no method has yet been developed for acrolein-specific trapping detection.

This present study aimed thus at establishing a novel and user-friendly HPLC-DAD approach for the rapid screening of acrolein scavengers in complex extracts. A multicomponent mixture of ten commercial standards was employed to mimic the chemical diversity of herbal matrices. Pre-column incubation with three acrolein concentrations was performed to support the identification of effective scavengers. The method’s effectiveness was finally assessed by comparing the results with those obtained through conventional individual assessments of each compound of the mixture.

## Method details

### Materials and reagents

Acrolein solution (5 mg/mL in methanol) was obtained from AVANTOR (Dr. Ehrenstorfer, LCG, DRE-GA09010384ME), aliquoted into various volumes without further dilution upon receipt, and stored at −20 °C for up to two weeks before use. HPLC grade methanol, acetonitrile and trifluoroacetic acid (TFA), dimethylsulfoxide (DMSO), hydrochloric acid (HCl), disodium hydrogen phosphate, and sodium dihydrogen phosphate were also purchased from AVANTOR (VWR Chemicals). 2,4-Dinitrophenylhydrazine hydrochloride (DNPH), aminoguanidine hydrochloride, esculin, piceid and phloretin were obtained from TCI Chemicals. Catechin, coumaric acid, epicatechin and phloridzin and were purchased from Sigma-Aldrich. Protocatechuic acid, and rutin were supplied by Alfa Aesar. Resveratrol was acquired from Cayman Chemical.

Ultrapure water (18.2 MΩ.cm) was produced in situ with an ELGA Purelab Ultra apparatus. Phosphate buffer (PB, 50 mM) was prepared by dissolving 545 mg of disodium hydrogen phosphate and 139.3 mg of sodium dihydrogen phosphate in ultrapure water, adjusting to pH 7.4, and bringing the final volume to 100 mL. The solution was stored at 4 °C in the dark for up to one week, with pH verified and corrected, if necessary, before use.

Chromatographic analyses were performed using an Agilent LC1220 system equipped with a diode array detector (DAD, Agilent G7117C). Peak area values were obtained from chromatograms using Agilent Data Analysis (v2.7) software. All experiments were performed in triplicate. Subsequent data processing was carried out with GraphPad Prism (v8). Values are expressed as mean ± standard error of the mean (SEM). Statistical analyses were performed using Dunnett’s test, with p < 0.05 considered indicative of a significant difference.

### Pre-column incubation approach coupled with HPLC-DAD analysis for fast screening of acrolein scavengers in complex matrices

This new application of HPLC-DAD can be divided into two main steps. The first involves sample preparation and incubation of the mixture in the presence of acrolein, while the second consists of a chromatographic analysis to detect decreases in the signal intensities of active compounds.

#### Sample preparation and incubation of the multicomponent mixture with acrolein

Incubation solutions (200 µL) were prepared by combining 100 µL of PB (50 mM, pH = 7.4), 50 µL of a ten-component standard mixture containing protocatechuic acid, esculin, catechin, epicatechin, coumaric acid, piceid, rutin, phloridzin, resveratrol, and phloretin (4 mM each, yielding a final concentration of 1 mM in the incubation solution), and 50 µL of a methanol-diluted acrolein solution.

Diluted acrolein solutions were prepared by aliquoting the pure stock solution (5 mg/mL) into Eppendorf tubes stored at −20 °C until use. Methanol was then added extemporaneously to reach the target acrolein concentrations, expressed relative to the overall incubation mixture containing PB and the multicomponent standard. Specifically, 478/22, 444/56, and 390/110 µL of methanol/acrolein stock solution were combined to obtain working solutions for experiments at 1, 2.5, and 5 mM acrolein, respectively. All samples were incubated at 37 °C for 90 min in a water bath, then diluted fourfold with ultrapure water and filtered through a 0.22 µm PTFE membrane filter (Phenomenex CLARIFY PTFE, 13 mm, 0.22 µm) prior chromatographic analyses. A control solution containing no acrolein (0 mM) was prepared under the same conditions as the other samples.

#### Chromatographic analyses

Chromatographic analyses were performed by reverse-phase high-performance liquid chromatography (RP-HPLC) on a Phenomenex KINETEX® EVO C_18_ column (2.1 × 150 mm, 5 µm). The mobile phase consisted of solvent A (water with 0.1 % TFA) and solvent B (acetonitrile with 0.1 % TFA). Elution was carried out using the following gradient (time in minutes, %B): 0–2 min, 8 % B; 2–22.5 min, 8–25 % B; 22.5–32.5 min, 30–43 % B; 32.5–42.5 min, 100 % B. The flow rate was 0.25 mL/min and the injection volume was 5 µL. Detection was performed using DAD detection, with monitoring wavelengths selected according to the UV absorption maxima of each compound. Results were expressed as a percentage relative to the control solution containing no acrolein (0 mM).

### Conventional method for evaluating acrolein-trapping capacity of isolated compounds

This protocol, allowing the evaluation of the acrolein-trapping potential of isolated compounds, was carried out based on the protocol of Zhu et al [4], with slight modifications. The procedure can also be divided into two main parts: the first involves sample preparation and incubation of the isolated compounds in the presence of acrolein, and the second consists of a chromatographic analysis to measure the remaining acrolein following a reaction with DNPH.

#### Sample preparation and incubation of the isolated compounds in the presence of acrolein

A 5 mM solution of DNPH was freshly prepared each day by dissolving 3.37 mg of DNPH in 2.5 mL of acetonitrile and 375 μL of 1 M HCl. The solution was sonicated for 2 min, stored at 4 °C, and vortexed before use. A 1 mM acrolein solution in 50 mM PB (50 mM, pH = 7.4) was also prepared daily by mixing 10 μL of acrolein stock solution (5 mg/mL) with 882 μL of PB. Aliquots were stored frozen and thawed with vortexing prior to use. Solutions of protocatechuic acid, esculin, catechin, epicatechin, coumaric acid, piceid, rutin, phloridzin, resveratrol, phloretin, and aminoguanidine (1 mM in PB containing 10 % DMSO) were prepared individually on a daily basis from 60 mM DMSO stock solutions. Briefly, 10 µL of each stock solution was mixed with 50 µL DMSO to obtain a 10 mM intermediate solution. This solution was further diluted by mixing 10 µL with 90 µL PB to yield a 1 mM working solution. A blank solution consisting of PB with 10 % DMSO, was also daily prepared by mixing 90 μL of PB with 10 μL of DMSO. Aminoguanidine was used as the positive control.

For incubation, 50 μL of the 1 mM acrolein solution in PB was mixed with 50 μL of either the blank solution or the test compound solution (1 mM in PB/10 % DMSO). The mixtures were incubated for 90 min at 37 °C in a water bath. After incubation, 60 μL of the 5 mM DNPH solution was added to each sample, which were then incubated for an additional hour at 37 °C. Following incubation, samples were diluted twofold with water and filtered through a 0.22 µm PTFE membrane filter (Phenomenex CLARIFY PTFE, 13 mm, 0.22 µm) prior to HPLC injection.

#### Chromatographic analyses

Chromatographic analysis was performed on the same Phenomenex KINETEX® EVO C^18^ column (2.1 × 150 mm, 5 µm) using the mobile phases described above. The gradient was set as follows (time in minutes, %B): 0–3 min, 35 % B; 3–23 min, 35–45 % B. The flow rate was set to 0.25 mL/min, the injection volume was 5 µL, and detection was carried out at 375 nm. Results were expressed as the percentage of residual acrolein, calculated relative to the blank sample containing acrolein only.

### Method validation

**Fig. 1:**
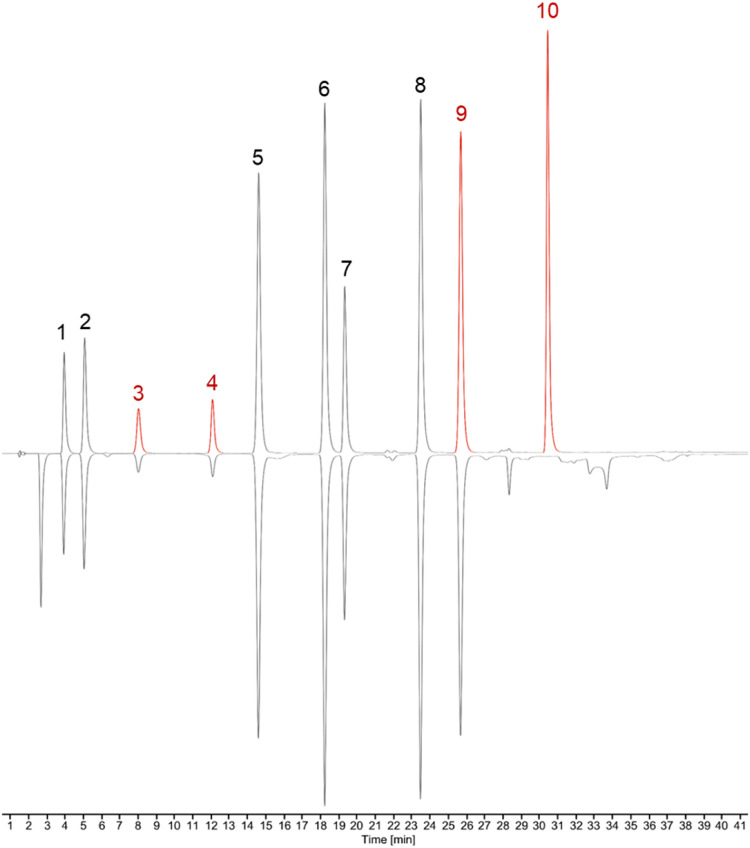
Chromatographic profile of the ten-component mixture, with detection at a wavelength of 280 nm. The upper chromatogram corresponds to the negative control (0 mM acrolein), while the lower chromatogram represents the mixture following pretreatment with 5 mM acrolein. 1: protocatechuic acid, 2: esculin, 3: catechin, 4: epicatechin, 5: coumaric acid, 6: piceid, 7: rutin, 8: phloridzin, 9: resveratrol, 10: phloretin. Acrolein-trapping compounds are highlighted in red.

The proposed method aims at directly identifying compounds capable of trapping acrolein within a complex extract. This approach relies on incubating the investigated matrix with a range of acrolein concentrations, followed by HPLC-DAD analysis. Reactive constituents are expected to exhibit specific signal decreases, whereas inactive compounds remain unaffected. In line with this principle, selective peak reductions were observed for a model mixture containing ten phenolic compounds, including protocatechuic acid, esculin, catechin, epicatechin, coumaric acid, piceid, rutin, phloridzin, resveratrol, and phloretin, as presented in Fig. 1.

**Fig. 2:**
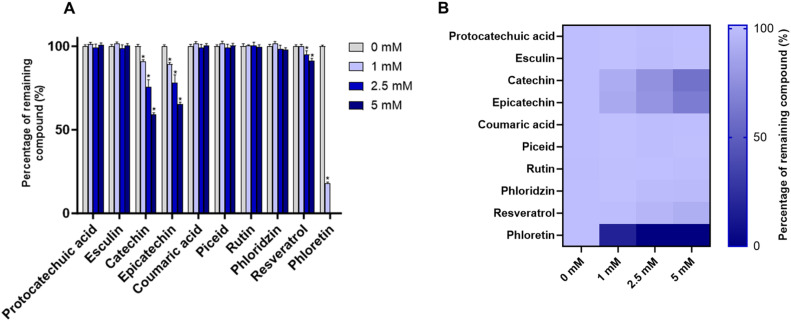
(A) Percentage of remaining compounds after incubation of the mixture with various concentrations of acrolein (0, 1, 2.5, and 5 mM). Data are presented as means ± SEM (*n* = 3). * *p* < 0.05 vs. control (acrolein= 0 mM); (B) Heat map representation of the percentage of remaining compounds after incubation of the mixture with acrolein.

Incubations were performed with acrolein concentrations of 1, 2.5, and 5 mM and compared with control samples lacking acrolein (0 mM). The corresponding quantitative data, detailing the percentage of remaining compounds across all tested concentrations are summarized in Fig. 2.

Interestingly, four compounds were identified as reactive: phloretin, catechin, epicatechin, and, to a lesser extent, resveratrol. Significant signal decreases (*p* < 0.05) were indeed observed, confirming their trapping ability. Phloretin proved to be particularly reactive, showing a marked reduction upon incubation with 1 mM acrolein (- 82.1 %) and complete signal loss at higher concentrations, demonstrating its outstanding efficiency. Catechin and epicatechin also showed notable activity. Exposure to 1 mM acrolein led to an approximate 10 % decrease in their signals (*p* < 0.05), while at 5 mM, more pronounced reductions of 40.6 % and 34.7 % were measured, respectively. Resveratrol exhibited a more modest trapping capacity, with small but significant decreases only detected at 2.5 and 5 mM (5.1 % and 8.5 %, respectively). Conversely, the remaining six compounds, namely protocatechuic acid, esculin, coumaric acid, piceid, rutin, and phloridzin, displayed unnoticeable reactivity.

**Fig. 3:**
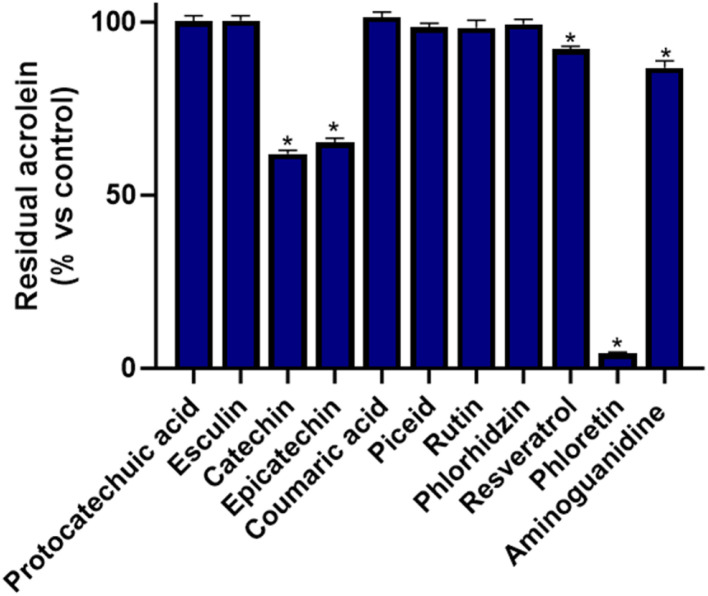
Percentage of residual acrolein after incubation with individual compounds. Data are presented as means ± SEM (*n* = 3). * *p* < 0.05 vs. control (acrolein alone).

To further validate the method and assess its predictive power and efficiency, all constituents of the simulated extract were individually evaluated for their acrolein scavenging properties using a previously reported protocol [4]. Importantly, while the novel method described above is capable of discriminating between the activities of compounds within a complex matrix, this second experiment focuses solely on the activity of isolated compounds. As shown in Fig. 3, the results closely matched the predictions obtained with our method. Phloretin displayed the highest scavenging activity, markedly surpassing the positive control, aminoguanidine. As expected, strong effects were also observed for epicatechin and catechin, which were also significantly superior to the positive control (*p* < 0.05). Resveratrol exhibited moderate scavenging capacity, consistent with the earlier observations, whereas the six other constituents (protocatechuic acid, esculin, coumaric acid, piceid, rutin, and phloridzin) showed negligible activity under the experimental conditions employed.

Validation of these findings was finally achieved through comparison with literature data. In agreement with our results, the strong acrolein-trapping capacity of phloretin has been widely reported [4,10], along with the good activities of epicatechin [4], and resveratrol [5]. The obtained results are also consistent with previous structure-activity relationship considerations. The presence of resorcinol motifs and, ideally, phloroglucinol motifs are indeed known to enhance the reactivity of phenolic compounds toward acrolein [4,6]. In both cases, the strong electron-donating effect of hydroxyl groups in the *meta* configuration is thought to generate electron-rich centers at the unsubstituted carbon sites, thereby facilitating electrophilic substitution reactions with electrophiles such as reactive carbonyl species. The presence of a phloroglucinol motif in phloretin, as well as a resorcinol motif in resveratrol, catechin, and epicatechin, appears therefore to account for the observed trapping activities of these compounds. Conversely, the lack of activity of derivatives such as protocatechuic acid, esculin, or coumaric acid is consistent with the absence of such patterns. For rutin, studies have shown that flavonols, including its aglycone form, quercetin, display little to no ability to trap acrolein [4]. Finally, the inactivity of phloridzin, a glycoside of phloretin, can be attributed to the glycosylation of the hydroxyl group at position 2 of its A ring, which is known to reduce activity [11]. A similar effect is likely observed for piceid, a glycoside of resveratrol.

Overall, these results validate the acrolein-scavenging properties of the four predicted active compounds: phloretin, epicatechin, catechin, and resveratrol. Additionally, these experiments also confirm that the novel detection method not only enables the identification of active constituents within complex mixtures but also offers a reliable ranking of their relative trapping efficiencies. Based on these results, this approach holds strong potential for accelerating the discovery of acrolein-trapping compounds, and its broader application to diverse plant extracts could yield important and impactful insights.

## Limitations

### Sample preparation and incubation with acrolein

As indicated by the manufacturer, acrolein is a relatively unstable product with a short shelf life. Therefore, it is recommended to perform the experiments without delay upon receipt of the product. In the present experiment, aliquots were prepared immediately after opening the vial and were directly stored at −20 °C for subsequent use. A satisfactory stability of the aliquots was observed for a storage period of up to two weeks.

For the incubation of extracts with acrolein, performing the assay at pH 7.4 and 37 °C is recommended to better mimic biological conditions and obtain results that are physiologically more relevant. The quantitative ratios between the extract to be evaluated and acrolein proposed in this study are expected to cover a wide range of reactivity. An initial acrolein concentration of 5 mM (final concentration in the incubation solution) applied to an extract at a concentration of 5 mg/mL (final concentration) is recommended for a first evaluation, enabling a strong indication of each component’s trapping capacity. Additional acrolein concentrations, such as 1 and 2.5 mM, may then be applied to more effectively rank the relative efficiency of different reactive constituents. Nevertheless, an adjustment of the acrolein concentration may be necessary in cases of low or very high scavenging activity of the extract. Final acrolein concentrations above 5 mM (e.g., 10 mM or 20 mM) or below 1 mM (e.g., 0.25 mM or 0.5 mM) could be respectively required in these situations.

The incubation time (90 min) was selected based on preliminary experiments with both shorter and longer durations. This period was considered optimal to achieve pronounced reaction rates for the active compounds under study. However, this choice may limit the detection of very slowly reacting constituents, and a longer incubation could be considered to identify additional scavengers. Incubation periods of 120, 180, or 240 min could then be considered in cases of insufficient reactivity of the evaluated compounds. It should be noted that, from an interventional perspective, compounds that react too slowly are nevertheless unlikely to exert meaningful biological effects, and their limited trapping capacity might be physiologically irrelevant.Of note, a four-fold dilution of the sample in ultrapure water is recommended at the end of the incubation. A 0.22 µm membrane filtration step is also advised before HPLC injection to maintain optimal chromatographic performance and prevent analytical issues.

### Chromatographic conditions

This publication describes a general method allowing the efficient and direct detection of acrolein scavengers in a complex matrix. However, its application to the analysis of a given plant extract will require, as with any HPLC analysis, adaptation and optimization of the chromatographic conditions (column type, mobile phase composition, gradient,…) according to the extract chemical composition. For guidance on method optimization and adaptation to complex plant matrices, users may refer to several key resources on HPLC method development and phytochemical analysis [[12], [13], [14]]. Regarding the analysis of extracts treated with acrolein, particular attention should also be paid to the retention times of the adducts formed between acrolein and the scavenging compounds. These additional peaks can potentially cause coelution or peak splitting. In such rare and isolated cases, further chromatographic optimization may be required.

## Ethics statements

None

## Declaration of generative AI and AI-assisted technologies in the writing process

Authors acknowledge the use of ChatGPT 4.0 for grammar checking and language refinement. The content, data analysis, and scientific interpretations are solely our own.

## CRediT authorship contribution statement

**Andy Zedet:** Investigation, Methodology, Supervision. **Alison Aebischer:** Investigation, Methodology, Formal analysis. **Marc Pudlo:** Validation, Writing – review & editing. **Luca Marchisio:** Validation. **Samba Fama Ndoye:** Investigation. **Corine Girard:** Writing – review & editing, Project administration. **François Senejoux:** Conceptualization, Supervision, Writing – original draft.

## Declaration of competing interest

The authors declare that they have no known competing financial interests or personal relationships that could have appeared to influence the work reported in this paper.

## Data Availability

Data will be made available on request.
